# Prevalence of pediatric acute-onset neuropsychiatric syndrome (PANS) in children and adolescents with eating disorders

**DOI:** 10.1186/s40337-022-00707-6

**Published:** 2022-12-13

**Authors:** Marya Aman, Jennifer S. Coelho, Boyee Lin, Cynthia Lu, Clara Westwell-Roper, John R. Best, S. Evelyn Stewart

**Affiliations:** 1 BC Children’s Hospital Research Institute, Vancouver, BC Canada; 2 Department of Psychiatry, Faculty of Medicine, University of British Columbia, Vancouver, BC Canada; 3 Provincial Specialized Eating Disorders Program for Children and Adolescents, BC Children’s Hospital, Vancouver, BC Canada; 4 BC Mental Health and Substance Use Services, Vancouver, BC Canada

**Keywords:** Pediatric acute onset neuropsychiatric syndrome, Pediatric autoimmune neuropsychiatric disorders associated with streptococcal infections, Eating disorder, Prevalence

## Abstract

**Background:**

Pediatric obsessive–compulsive disorder (OCD) and eating disorder symptoms frequently overlap, clouding diagnostic certainty and hypothesized etiologic factors. Pediatric acute-onset neuropsychiatric syndrome (PANS) is defined by abrupt emergence of core obsessive–compulsive behaviours and/or food restriction with concurrent, ancillary cognitive and behavioral symptoms. Inflammatory and immune processes have putative roles in both PANS and a related described condition with cardinal obsessive–compulsive or tic symptoms, known as pediatric autoimmune neuropsychiatric disorders associated with streptococcal infection (PANDAS). While prevalence of PANS and PANDAS has been examined in tic, movement disorder and OCD populations, this has not yet been systematically examined in a pediatric eating disorder sample.

**Objectives:**

To identify the lifetime prevalence of those meeting PANS and/or PANDAS criteria within a pediatric eating disorder cohort.

**Methods:**

Convenience sampling method was utilized to select consecutive youth (ages 8–18-years) presenting to an interdisciplinary pediatric eating disorder subspecialty program with a confirmed eating disorder and completed parent-report PANS/PANDAS questionnaire (n = 100). A parent-reported measure was used to establish lifetime prevalence rates for PANS and PANDAS. Descriptive and exploratory comparative analyses were conducted between PANS and non-PANS groups. Continuous measures were analyzed using two-tailed independent sample t-tests and categorical measures were analyzed using two-tailed Fisher’s exact tests.

**Results:**

Among participants, 52% (n = 52) met PANS criteria and 0% (n = 0) met PANDAS diagnostic criteria. Core, abrupt-onset PANS symptoms included both food restriction and obsessive–compulsive symptoms in 63.5% (n = 33), food restriction only in 25% (n = 13), and obsessive–compulsive symptoms only in 11.5% (n = 6) of participants. In comparison to those who did not meet PANS criteria, those in the PANS subgroup were less likely to be male and more commonly prescribed a selective serotonin reuptake inhibitor medication. Significant group differences did not emerge for onset age, body mass index, eating disorder type or comorbid psychiatric/medical/autoimmune illness.

**Conclusion:**

Lifetime prevalence of symptoms in keeping with PANS diagnostic criteria within a pediatric eating disorder cohort was notably higher than that previously reported in OCD or tic disorder cohorts. The overlap between starvation effects and ancillary PANS symptoms may challenge the practical utility of this putative syndrome within the eating disorder population.

**Supplementary Information:**

The online version contains supplementary material available at 10.1186/s40337-022-00707-6.

## Plain English summary

The connections and overlap between eating disorders and obsessive–compulsive disorder (OCD) are complicated and not fully understood. A syndrome described in the past decade (pediatric acute-onset neuropsychiatric syndrome; PANS) is characterized by a sudden, dramatic onset of food restriction and/or obsessive–compulsive symptoms in combination with several other behavioural and cognitive changes; a related condition is associated with sudden onset obsessive–compulsive symptoms or tics after streptococcal infection (pediatric autoimmune neuropsychiatric disorders associated with streptococcal infection; PANDAS). Rates of PANS and PANDAS have been reported in OCD and tic populations but not in eating disorders. We set out to screen a group of youth at a pediatric eating disorders program for lifetime symptoms of PANS and PANDAS. Among 100 eating disorder affected-youth in this study, approximately half (52%) met criteria for PANS, and none met criteria for PANDAS. However, the overlap between several PANS diagnostic criteria items and effects of starvation on both cognition and behaviour clouds the potential utility of this putative subtype within eating disorders populations.

**Supplementary Information:**

The online version contains supplementary material available at 10.1186/s40337-022-00707-6.

## Background

The current study set out to examine the overlap between symptoms of eating disorders, and sudden onset obsessive–compulsive symptoms. Obsessive–compulsive disorder (OCD) has a lifetime prevalence rate of 1.3% in the general population, peaking in preadolescence and early adulthood [1]. Eating disorders are also common in youth and young adults, representing the third most common chronic illness in adolescents [2]. Eating disorders are associated with a high risk of mortality, functional impairment, and high rate of comorbidity [3, 4]. Single studies report a high proportion of individuals with eating disorders presenting with co-occurring anxiety disorders and OCD, including reports of an estimated 64% of individuals with eating disorders diagnosed with at least one anxiety disorder, and 41% meeting criteria for OCD [5]. Metanalysis indicates a lower proportion of individuals with co-occurring eating disorders and OCD, with lifetime prevalence estimates of comorbid OCD in eating disorders of 13.9% [6]. In addition to the frequent co-occurrence of OCD and eating disorders, there is emerging data to support a genetic correlation with anorexia nervosa (AN), and OCD, suggesting a shared etiology [7–9]. The Cross-Disorder Group of the Psychiatric Genomics Consortium recently reported that based on genome-wide genetic correlations, there is close correlation between OCD and AN [10].

Within pediatric OCD, a subgroup of youth present with a dramatic, sudden onset of symptoms that appear to follow a streptococcal infection [11]. This syndrome has been labelled “pediatric autoimmune neuropsychiatric disorders associated with streptococcal infection” (PANDAS) and working criteria for the diagnosis of this syndrome are established [11]. Debates about the validity and utility of the PANDAS criteria led to the development of modified criteria for a new clinical syndrome identified as “pediatric acute-onset neuropsychiatric syndrome” (PANS) [12]. There are various proposed etiologies for PANS, including postinfectious sequelae, and genetic and immunologic disorders [13]. When group A streptococcal (GAS) infection precedes OCD symptoms and/or tics the diagnosis is more consistent with PANDAS.

Whereas the initial syndrome of PANDAS focused on abrupt onset of OCD and/or tics, the central criteria for PANS were changed to include sudden onset of “eating restriction or anorexic behaviors” and/or OCD [12, p. 3]. The inclusion of eating-related behaviors in the proposed PANS criteria were based on clinical observations of acute onset of restrictive eating behavior that was first described in a case study of a prepubescent youth with weight gain fears as a result of body dysmorphia, and symptoms temporally related to a GAS infection [14]. Since then, additional case studies have described youth with sudden onset obsessional fears of contamination, fears of poisoning, choking, or vomiting, and/or sensory concerns based on texture, taste or smell [15]. In some cases, body image distortions were also present although emerging later in the symptom course [15]. A separate case report also highlighted a case of anorexia nervosa and sudden onset OCD symptoms that were preceded by a GAS infection [16]. However, the notion of an abrupt-onset version of AN (PANDAS-AN) has been controversial, stemming from difficulties in diagnosing cases, relatively low prevalence, and calls to refine diagnostic criteria for this clinical condition [17]. There is limited research in the field of eating disorders about PANDAS-AN; however, there has recently been some attention to the overlap between PANS/PANDAS and avoidant/restrictive food intake disorder (ARFID) [18].

To date the prevalence of PANS/PANDAS has been evaluated in pediatric patients with tic disorder (n = 80, 11% abrupt symptom onset), children at a movement disorders clinic (n = 284, 1% PANS), and youth at an outpatient OCD clinic (n = 136, 5% PANS/PANDAS) [19–21]. To our knowledge, no study has screened an eating disorder population for PANS/PANDAS. Understanding the rates of prevalence of PANS/PANDAS in this population has direct clinical implications as this subgroup has different proposed etiologies and treatments. The primary objective of this study was to assess the prevalence of PANS and PANDAS diagnostic symptoms in a pediatric eating disorder sample. The secondary objective was to describe the clinical characteristics of those presenting with PANS/PANDAS characteristics in comparison with those not meeting criteria for PANS/PANDAS.

## Methods

### Diagnostic assessments

Physician-referred assessments were conducted between November 2017 and August 2019 at British Columbia Children's Hospital Provincial Specialized Eating Disorders Program for Children and Adolescents (for details about this program, please refer to [22]. Assessments comprise of a thorough medical and psychiatric history, as well as a physical exam. Eating disorder diagnosis is established through clinical assessment with a psychologist or psychiatrist specialized in eating disorders.

### Study measures and data collection

The sampling method used in this study is convenience sampling. The ‘PANS and PANDAS questionnaire’ ([21]; see Additional file 1) is a parent-report measure, comprising an initial question as to whether their child ever had rapid, sudden onset (within hours, or one to two days) of food refusal, obsessions or compulsions, or tics. Those responding in the affirmative are asked to complete the remaining questions itemizing PANS and PANDAS criteria (see Table 1) using proposed diagnostic criteria by Swedo et al. [12]. The measure was developed based on proposed diagnostic criteria for a pediatric OCD study by our group [21]. The psychometric properties of this measure have not yet been established.

**Table 1:** Proposed diagnostic criteria for pediatric autoimmune neuropsychiatric disorders associated with streptococcal infections (PANDAS) and pediatric acute-onset neuropsychiatric syndrome

I.	Diagnostic criteria for pediatric autoimmune neuropsychiatric disorders associated with streptococcal infection (PANDAS)^a^:
	a. Presence of obsessive–compulsive and/or tics disorder
	b. Pediatric onset symptoms must begin between 3 years—puberty
	c. Abrupt onset of symptoms or dramatic symptom exacerbation with a saw-tooth course
	d. Association with a confirmed streptococcal infection
	e. Association with other neuropsychiatric symptoms (e.g. choreiform movements)
II.	Diagnostic criteria tor pediatric acute-onset syndrome (PANS)^b^:
	a. Abrupt and dramatic onset of OCD symptoms or severe restriction of food intake
	b. Concurrent and sudden onset of at least two of the following symptoms:
	i. Anxiety
	ii. Emotional lability/depression
	iii. Irritability
	iv. Aggression and/or oppositional behaviors
	v. Behavioral (developmental) regression
	vi. Deterioration in school performance
	vii. Sensorimotor abnormalities
	viii. Somatic signs and symptoms^c^
	c. Symptoms cannot be better explained by a known neurological or medical condition^d^

*OCD* obsessive–compulsive disorder

^a^As proposed by Swedo et al.[11]

^b^As proposed by Swedo et al.[12]

^c^Such as sleep disturbances, enuresis, or urinary frequency

^d^Such as Sydenham chorea, systemic lupus erythematosus, or Tourette’s disorder

Additional data were collected via chart review regarding gender, age at assessment and at eating disorder onset, eating disorder diagnosis, psychiatric and medical comorbidities, medications, and body mass index (BMI). Autism spectrum disorder and tic disorder were not specifically screened for as comorbidities, but were noted in the assessment report if previously flagged by a referring source. BMI was calculated using height measured at assessment (or, if not available at assessment, within 2 months of assessment), and is reported as percent median BMI (% mBMI) according to World Health Organization reference values [23].

### Eligibility

Criteria for study eligibility included the following: eating disorder diagnosis according to the DSM-5 criteria [24], and completion of the PANS and PANDAS questionnaire by a parent/guardian.

### Data analyses

Participants were categorized into those who endorsed PANS/PANDAS criteria, and those who denied symptoms of PANS/PANDAS.

Exploratory comparative analyses were conducted between PANS and non-PANS groups. Continuous measures were analyzed using two-tailed independent sample t-tests; equal variances were assumed given non-significance of Levene’s Tests (*p* > 0.05). Two-tailed Fisher’s exact tests were used for categorical measures. For all analyses, the statistical threshold was set at α = 0.05. Given that the comparative analyses were exploratory, multiple-testing correction was not conducted. The statistical significance for variables with infrequent response categories (less than 5% of the total population) was not reported to reduce the overall number of analyses and allow for better description of the PANS vs non-PANS groups. SPSS [25] was used for all data analyses.

## Results

**Fig. 1:**
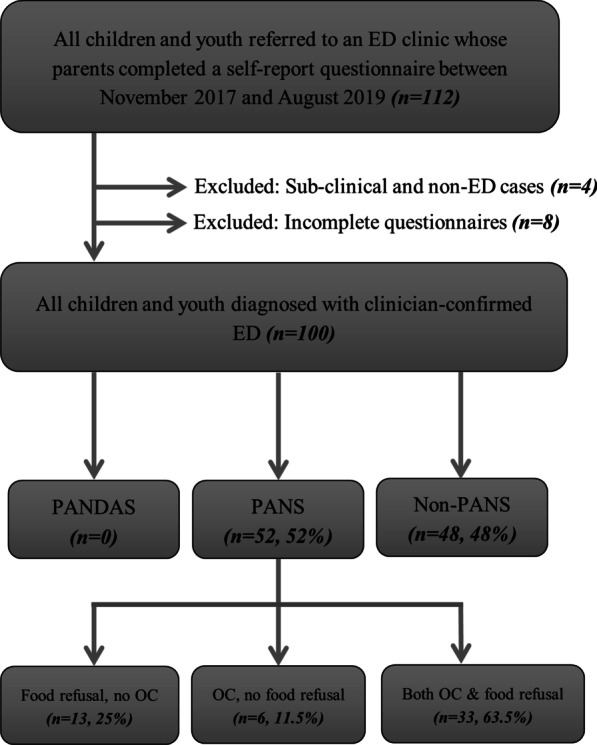
Sampling methodology used to determine prevalence of pediatric acute-onset neuropsychiatric syndrome (PANS) and pediatric autoimmune neuropsychiatric disorders associated with streprotococcal infections (PANDAS) in a pediatric eating disorder program. ED, eating disorder; PANS, pediatric acute-onset neuropsychiatric syndrome; PANDAS, pediatric autoimmune neuropsychiatric disorders associated with streptococcal infections; OC, obsessions compulsions

In total, 100 youth met inclusion criteria, comprising 79% (n = 79) cis-gender females, 20% (n = 20) cis-gender males, and 1% (n = 1) transgender youth, ranging between 8 and 18 years of age (mean age 14.19, SD = 2.18). Based on parental responses on the questionnaire, 52% met criteria for PANS (n = 52) and none met criteria for PANDAS (Fig. 1).

Within the PANS group, 63.5% (n = 33) had both abrupt onset obsessive–compulsive symptoms and eating restriction, 11.5% (n = 6) had abrupt onset obsessive–compulsive symptoms without eating restriction and only 25% (n = 13) had abrupt onset eating restriction without obsessive–compulsive symptoms.

### PANS versus non-PANS subgroups

There was a smaller proportion of males in the PANS group than in the non-PANS group (11.5% vs. 29.2% of total sample, respectively; p = 0.016). There were no significant differences between the PANS group and non-PANS group with respect to mean age at eating disorder symptom onset, eating disorder type, or %mBMI (Table 2). Nor were there significant group differences with respect to psychiatric or medical comorbidities. Prescribed selective serotonin reuptake inhibitors (but not other medications) were found to be more common in the PANS group than non-PANS group (23.1% vs. 8.3%, *p* = 0.045).

**Table 2:** Demographic characteristics, comorbidities, medications and parent reported symptoms for pediatric acute-onset neuropsychiatric syndrome subgroup in pediatric eating disorders population

	PANS group (n = 52)	Non-PANS group (n = 48)	t value^a^	*df*^a^	*P* value^a,b^
*Demographic characteristics*					
Gender, number male (%)	6 (11.5)	14 (29.2)			0.016
Symptom onset, mean age (SD)	13.17 (1.96)	12.79 (2.75)	0.803	98	0.131
Percent of median BMI for age, mean (SD)^c^	84.79 (13.66)	87.31 (13.90)	− 0.607	98	0.366
*Type of eating disorder*					
AN (%)	35 (67.3)	27 (56.3)			0.305
BN (%)	2 (3.8)	4 (8.3)			*
ARFID (%)	10 (19.2)	13 (27.1)			0.476
Other specified feeding or eating disorder (%)	1 (1.9)	1 (2.1)			*
Unspecified eating disorder (%)	4 (7.7)	3 (6.3)			*
*Co-occurring diagnoses*					
Mood disorder (%)^d^	9 (17.3)	5 (10.4)			0.394
Anxiety disorder (%)^e^	29 (55.8)	23 (47.9)			0.548
OCD (%)	5 (9.6)	3 (6.3)			0.717
Tic disorder (%)	1 (1.9)	0			*
ADHD (%)	5 (9.6)	6 (12.5)			0.754
Neurodevelopmental disorder (%)^f^	1 (1.9)	3 (6.3)			*
Somatic symptom disorder (%)	1 (1.9)	1 (2.1)			*
Autoimmune disorder (%)^g^	2 (3.8)	1 (2.1)			*
*Medications*					
SSRI (%)^h^	12 (23.1)	4 (8.3)			0.045
NDRI (%)^i^	1 (1.9)	0			*
ADHD medication (%) ^j^	0	2 (4.2)			*
Antipsychotic (%) ^k^	5 (9.6)	2 (4.2)			0.439
Sleep Aid (%)^l^	2 (3.8)	1 (2.1)			*
Benzodiazepine (%)^m^	5 (9.6)	2 (4.2)			0.439
Antibiotic (%)^n^	0	1 (2.1)			*
Anti-inflammatory/Steroid (%)^o^	1 (1.9)	2 (4.2)			*
*Parent reported outcomes*					
Abrupt onset of either:					
Obsessions or compulsions (%)	6 (11.5)	2 (4.2)			0.272
Food refusal (%)	13 (25.0)	5 (10.4)			0.071
Both obsessions or compulsions and food refusal (%)	33 (63.5)	1 (2.1)			< 0.001
Tics (%)	10 (19.2)	3 (6.3)			0.054

*PANS* pediatric acute-onset neuropsychiatric syndrome, *BMI* body mass index, *AN* anorexia nervosa, *BN* bulimia nervosa, *ARFID* avoidant restrictive food intake disorder, *MDD* major depressive disorder, *GAD* generalized anxiety disorder, *OCD* obsessive compulsive disorder, *ADHD* attention deficit hyperactivity disorder, *SSRI* selective serotonin reuptake inhibitor, *NDRI* norepinephrine dopamine reuptake inhibitor

*Statistical significance was not reported for variables with an n that is less than 5% of the total population

^a^Independent samples t-test, two-tailed was used for continuous measures. Equal variances were assumed, as Levene’s Test was not significant in any instance (*p* > 0.05)

^b^Fisher’s Exact Test, two−tailed was used for dichotomous measures

^c^Recorded BMI was missing from two patient charts in the non-PANS group

^d^Including major depressive disorder, unspecified depressive disorder

^e^Including generalized anxiety disorder, social anxiety disorder, posttraumatic stress disorder, panic disorder, unspecified anxiety disorder

^f^Including autism spectrum disorder, global developmental delay, learning disorder

^g^Including hyperthyroidism, diabetes mellitus, asthma

^h^Including sertraline, fluoxetine, escitalopram, vortioxetine

^i^Including bupropion

^j^Including guanfacine, methylphenidate (biphentin and concerta)

^k^Including olanzapine, quetiapine, risperidone

^l^Including trazodone, melatonin

^m^Including lorazepam, clonazepam

^n^Including erythromycin

^o^Including cyproheptadine, fluticasone

## Discussion

To the authors’ knowledge, this is the first study to assess prevalence rates of PANS and PANDAS within a pediatric eating disorder sample. Over half (52%) of new cases met PANS criteria and none met PANDAS criteria.

The prevalence rate reported in this study is much higher than previously reported PANDAS/PANS rates in other clinical pediatric populations. In a sample of 136 pediatric OCD patients, only 3% were found to meet PANS criteria, using the same parent report measure as the current study [21]. In the PANS subgroup in that study, 60% reported sudden food restriction and 100% reported abrupt obsessive–compulsive symptoms [21]. Low rates of PANS/PANDAS have also been reported in pediatric motor disorders, in which less than 1% of 284 patients met PANS criteria [20]. Similarly, in a sample of 80 children with tic disorders, none of the 11% reporting rapid/abrupt symptom onset met criteria for PANDAS; PANS diagnosis had not yet been described at the time of study [19].

It is important to note that the physical and mental consequences of food restriction overlap with some of the PANS ancillary criteria. As a result, there is a lack of specificity of the PANS criteria in an eating disorder population, which likely contributed to higher than expected observed prevalence of PANS in this population. Symptoms of starvation include anxiety, depression, irritability, and sleep disturbance [26], which are diagnostic criterion items for PANS and/or PANDAS. Furthermore, the preoccupation with food that is characteristic of the starvation state may have obsessional qualities that may present as obsessive–compulsive -type symptoms; however, differential diagnosis considerations for obsessive–compulsive symptoms in the context of eating disorders highlight that concerns or behaviors specific to food or weight do not warrant an additional diagnosis of OCD [24]. Therefore, the current version of the self-report measure to assess the presence of PANS criteria has challenges for use in an eating disorder setting and may have limited utility for defining a putative eating disorder subtype. Routine screening for PANS may occur in some pediatric medical/psychiatric settings (e.g., OCD clinics). However, the current results suggest the need for caution if screening for PANS in individuals with eating disorder diagnoses given a likelihood of false positives.

Limitations of this study include a reliance on self-reported measures of PANS/PANDAS symptoms, and a lack of context for how parents defined “sudden onset” of symptoms. This may have represented food refusal in the context of gradually increasing food restriction. Furthermore, semi-structured clinical interviews were used to establish eating disorder diagnoses, as opposed to validated diagnostic interviews such as the Eating Disorder Examination [27]. Additionally, the relatively small sample size restricts the potential for comparative and predictive analyses.

Future research to elucidate parents’ perceptions of a sudden onset of symptoms in the context of an eating disorder is warranted. Parents have previously reported slow recognition of eating disorder symptoms [28], which can impact parental interpretations about the nature of onset of food refusal or other symptoms. Obtaining both youth and parental self-report, as well as assessing parent report during or post-treatment, is also warranted to determine the impact on the proportion of participants who endorse sudden onset of symptoms. Further exploration of autoimmune disorder concurrence in the eating disorder population, and of the utility of salivary biomarkers of inflammation, as proposed by Westwell-Roper and Stewart [29], is also warranted to elucidate potential pathways to the emergence of PANS symptoms in the context of eating disorders. Data from a population-based cohort have suggested the importance of immunologic factors in eating disorders, with childhood infections associated with later onset of an eating disorder [30].

## Conclusion

The findings of this research study indicate that those who screened positive for PANS comprise 52% of a pediatric eating disorder sample. The majority (63.5%) of identified participants screening positive for PANS within this eating disorder sample had both abrupt-onset eating restriction and abrupt-onset obsessive–compulsive symptoms. The validity of PANS criteria in an eating disorder setting is challenged by the notable overlap between the starvation syndrome (characteristic of those with AN and other eating disorders) and PANS diagnostic criteria.

## Supplementary Information

**Additional file 1.** PANS and PANDAS questionnaire.

## Data Availability

The datasets used and/or analysed during the current study are available from the corresponding author on reasonable request.

## Abbreviations

PANS: Pediatric acute-onset neuropsychiatric syndrome
PANDAS: Pediatric autoimmune neuropsychiatric disorder associated with streptococcal infections
BMI: Body mass index
ED: Eating disorder
AN: Anorexia nervosa
BN: Bulimia nervosa
ARFID: Avoidant restrictive food intake disorder
OCD: Obsessive compulsive disorder
OC: Obsessions compulsions
MDD: Major depressive disorder
GAD: Generalized anxiety disorder
ADHD: Attention deficit hyperactivity disorder
SSRI: Selective serotonin reuptake inhibitor
NDRI: Norepinephrine dopamine reuptake inhibitor

## References

[1] Fawcett EJ, Power H, Fawcett JM (2020). Women are at greater risk of OCD than men: a meta-analytic review of OCD prevalence worldwide. J Clin Psychiatry.

[2] Campbell K, Peebles R (2014). Eating disorders in children and adolescents: state of the art review. Pediatrics.

[3] Klump KL, Bulik CM, Kaye WH, Treasure J, Tyson E (2009). Academy for eating disorders position paper: eating disorders are serious mental illnesses. Int J Eat Disord.

[4] Pallister E, Waller G (2008). Anxiety in the eating disorders: Understanding the overlap. Clin Psychol Rev.

[5] Swinbourne J, Hunt C, Abbott M, Russell J, St Clare T, Touyz S (2012). The comorbidity between eating disorders and anxiety disorders: prevalence in an eating disorder sample and anxiety disorder sample. Aust N Z J Psychiatry.

[6] Drakes DH, Fawcett EJ, Rose JP, Carter-Major JC, Fawcett JM (2021). Comorbid obsessive-compulsive disorder in individuals with eating disorders: an epidemiological meta-analysis. J Psychiatr Res.

[7] Anttila V, Bulik-Sullivan B, Finucane HK, Walters RK, Bras J, Consortium B (2018). Analysis of shared heritability in common disorders of the brain. Science.

[8] Cederlöf M, Thornton LM, Baker J, Lichtenstein P, Larsson H, Rück C (2015). Etiological overlap between obsessive-compulsive disorder and anorexia nervosa: a longitudinal cohort, multigenerational family and twin study. World Psychiatry.

[9] Mas S, Plana MT, Castro-Fornieles J, Gassó P, Lafuente A, Moreno E (2013). Common genetic background in anorexia nervosa and obsessive compulsive disorder: preliminary results from an association study. J Psychiatr Res.

[10] Genomic relationships, novel loci, and pleiotropic mechanisms across eight psychiatric disorders—ScienceDirect [Internet]. [cited 2022 Aug 31]. https://www.sciencedirect.com/science/article/pii/S0092867419312760. 10.1016/j.cell.2019.11.020 PMC7077032 31835028

[11] Swedo SE, Leonard HL, Garvey M, Mittleman B, Allen AJ, Perlmutter S (1998). Pediatric autoimmune neuropsychiatric disorders associated with streptococcal infections: clinical description of the first 50 cases. Am J Psychiatry.

[12] Swedo SE, Leckman JF, Rose NR (2012). From research subgroup to clinical syndrome: modifying the PANDAS criteria to describe PANS (pediatric acute-onset neuropsychiatric syndrome). Pediatr Ther.

[13] Orefici G, Cardona F, Cox CJ, Cunningham MW. Pediatric autoimmune neuropsychiatric disorders associated with streptococcal infections (PANDAS). In: Ferretti JJ, Stevens DL, Fischetti VA, editors. Streptococcus pyogenes: Basic Biology to Clinical Manifestations [Internet]. Oklahoma: University of Oklahoma Health Sciences Center; 2016. PMID: 26866234. 26866234

[14] Sokol MS, Gray NS (1997). Case study: an infection-triggered, autoimmune subtype of anorexia nervosa. J Am Acad Child Adolesc Psychiatry.

[15] Toufexis MD, Hommer R, Gerardi DM, Grant P, Rothschild L, D’Souza P (2015). Disordered eating and food restrictions in children with PANDAS/PANS. J Child Adolesc Psychopharmacol.

[16] Ajueze P, Shivakumar K, Saroka K, Shivakumar K, Amanullah S (2018). A complex case of anorexia nervosa associated with pediatric acute-onset neuropsychiatric disorder associated with streptococcal infection (PANDAS). Complex clinical conundrums in psychiatry: from theory to clinical management [Internet].

[17] Puxley F, Midtsund M, Iosif A, Lask B (2008). PANDAS anorexia nervosa—endangered, extinct or nonexistent?. Int J Eat Disord.

[18] Strand M, von Hausswolff-Juhlin Y, Welch E (2019). A systematic scoping review of diagnostic validity in avoidant/restrictive food intake disorder. Int J Eat Disord.

[19] Singer HS, Giuliano JD, Zimmerman AM, Walkup JT (2000). Infection: a stimulus for tic disorders. Pediatr Neurol.

[20] Kilbertus S, Brannan R, Sell E, Doja A (2014). No cases of PANDAS on follow-up of patients referred to a pediatric movement disorders clinic. Front Pediatr.

[21] Jaspers-Fayer F, Han SHJ, Chan E, McKenney K, Simpson A, Boyle A (2017). Prevalence of acute-onset subtypes in pediatric obsessive-compulsive disorder. J Child Adolesc Psychopharmacol.

[22] Coelho JS, Lee T, Karnabi P, Burns A, Marshall S, Geller J (2018). Eating disorders in biological males: clinical presentation and consideration of sex differences in a pediatric sample. J Eat Disord.

[23] de Onis M, Onyango AW, Borghi E, Siyam A, Nishida C, Siekmann J (2007). Development of a WHO growth reference for school-aged children and adolescents. Bull World Health Organ.

[24] American Psychiatric Association (2013). Diagnostic and statistical manual of mental disorders: DSM-5.

[25] IBM Spss Statistics for Macintosh (2013). Armonk.

[26] Keys A, Brozek J, Henschel A, Mickelsen O, Taylor HL (1950). The biology of human starvation.

[27] Fairburn CG, Cooper Z, O’Connor M (1993). The eating disorder examination. Int J Eat Disord.

[28] Ciao AC, Lebow J, VandenLangenberg E, Ohls O, Berg KC (2022). A qualitative examination of adolescent and parent perspectives on early identification and early response to eating disorders. Eat Disord.

[29] Westwell-Roper C, Stewart SE (2020). Commentary: neurobiology and therapeutic potential of cyclooxygenase-2 (COX-2) inhibitors for inflammation in neuropsychiatric disorders. Front Psych.

[30] Breithaupt L, Köhler-Forsberg O, Larsen JT, Benros ME, Thornton LM, Bulik CM (2019). Association of exposure to infections in childhood with risk of eating disorders in adolescent girls. JAMA Psychiatry.

