# Supplementary material for: Prevalence of pediatric acute-onset neuropsychiatric syndrome (PANS) in children and adolescents with eating disorders
Source: J Eat Disord. 2022 Dec 13;10:194. doi: 10.1186/s40337-022-00707-6 (PMC9749211; doi:10.1186/s40337-022-00707-6)
Supplement: Supplementary file 1 — Additional file 1. PANS and PANDAS questionnaire. [file 40337_2022_707_MOESM1_ESM.docx]

**Additional file 1**

Right now, or in the past, has your child ever had rapid, sudden onset (e.g., within hours or 1-2 days) of any of the following?

| Refusal of food/refusing to eat  (for example, a sudden refusal to eat that appears out of the blue) | No Yes I don’t know |
| --- | --- |
| Obsessions or compulsions | No Yes I don’t know  If yes – how old was your child (in years) when this first happened? ____ |
| Tics | No Yes I don’t know  If yes – how old was your child (in years) when this first happened? ____ |
| *If you answered no to the questions above – please skip the rest of this questionnaire*  **If you answered YES to any of the questions (i.e. your child has had rapid/sudden onset of obsessions/compulsions, tics, or refusal of food)…** | |
| …do the sudden bouts (periods) of food refusal, obsessions/compulsions or tics come and go over time? | No Yes I don’t know |
| …do the sudden bouts (periods) of food refusal, obsessions/compulsions or tics appear within weeks of a “strep” infection? | No Yes I don’t know |
| When the sudden food refusal, obsessions/compulsions or tics are present, does your child also seem more hyper than usual, or have any movement/coordination abnormalities? | No Yes I don’t know |
| When the sudden food refusal, obsessions/compulsions or refusal of food is present, does your child also have any of the following? (please check all that apply) | □Anxiety  □Moodiness or depression  □Irritability, aggression, and/or severe disobedience  □Acting younger than usual  □Worse school performance  □Abnormalities/changes in his/her movements or heightened senses  □Sleep problems, bedwetting, and/or frequent urination  □None of the above |
